# A Novel Open-Loop Current Sensor Based on Multiple Spin Valve Sensors and Magnetic Shunt Effect with Position Deviation Calibration

**DOI:** 10.3390/mi16080953

**Published:** 2025-08-19

**Authors:** Tianbin Xu, Tian Lan, Jiaye Yu, Yu Fu, Boyan Li, Tengda Yang, Ru Bai

**Affiliations:** 1 School of Electronics and Information, Hangzhou Dianzi University, Hangzhou 310018, China; tianbin.xu@hdu.edu.cn (T.X.); blueskylt180@126.com (T.L.); jayyu@hdu.edu.cn (J.Y.); yu.fu@hdu.edu.cn (Y.F.); tzlby529@163.com (B.L.); 221040081@hdu.edu.cn (T.Y.); 2 School of Information Science and Technology, Hangzhou Normal University, Hangzhou 311121, China

**Keywords:** current sensor, magnetic shunt effect, spin sensing technology, data fusion algorithm

## Abstract

To address the demands for wide-range and high-precision current measurement, this paper proposes a novel current sensor design that integrates spin sensing technology, magnetic shunt effect, and a multi-sensor data fusion algorithm. The spin valve sensors accurately detect the magnetic field generated by the signal current, while the soft magnetic shunt structure attenuates the magnetic field to a level suitable for the spin valve sensors. Consequently, the detection current range can be extended by 6.8 times. Using four spin valve sensors and data fusion with an averaging algorithm, the system can calibrate the errors caused by the displacement or tilt of the current-carrying wire. Experimental results demonstrate that the current sensor achieves a sensitivity of 61.6 mV/V/A, an excellent linearity of 0.55%, and robust measurement performance, as well as strong anti-interference capability. Our study offers a novel solution for high-precision, wide-range current measurement in applications such as those in new energy vehicle electronics and precision electric energy metering.

## 1. Introduction

Current sensors are extensively utilized in various fields such as automotive electronics, industrial control systems, medical equipment, and consumer electronics [1,2,3,4]. With the continuous progress of technology, the performance requirements of current sensors are becoming higher and higher. Currently, the main types of current sensors include fluxgate, optical fiber, Hall effect, current transformer, shunt, and magnetoresistive sensors [5]. Their characteristics vary depending on different application scenarios, such as AC/DC operation and the magnitude of the current. The fluxgate current sensors are known for their high detection accuracy and excellent temperature stability, and are suitable for scenarios that require high-precision DC or low-frequency AC measurements. However, their circuit design is complex, and the volume is large [6,7]. Although the fiber-optic current sensor has the advantages of high precision and anti-electromagnetic interference in high-voltage isolation or harsh EMI environments (e.g., power systems), the production process is complex and susceptible to temperature and vibration [8,9]. The Hall effect current sensors are favored for their compact size and low cost, and are used in cost-sensitive, space-constrained medium-current (ranging from several amperes to several hundred amperes) DC or AC measurements, but their sensitivity, bandwidth, and temperature drift must be carefully addressed during both design and application [10]. Current transformers can effectively provide electrical isolation, making them the standard solution for AC high-current measurements, but there are problems such as saturation, noise interference, and an inability to detect direct currents [11]. The shunt current sensors have a low cost and can measure large currents. They are prevalent in direct-connection DC high-current measurement applications. However, their measurement accuracy can be compromised by factors such as parasitic inductance, temperature, and other factors, and there are safety hazards due to significant heat generation at high currents [12]. The Rogowski coil exhibits fast response characteristics, making it particularly suitable for measuring high-frequency AC or transient pulse currents, but it can only measure alternating current (AC) or high-speed pulsed currents. Additionally, its signal processing circuit is relatively complex [13].

Spin sensors, like giant magnetoresistive (GMR), spin valve, and tunneling magnetoresistive (TMR) sensors, represent an advanced technology for current measurement. These sensors offer high sensitivity, low power consumption, and fast response, making them ideal for high-precision, miniaturized, wide-bandwidth DC/AC current sensing [14,15,16,17,18,19,20,21]. Many research works have used spin sensors to design current sensors. Behera, B., et al. prepared Co/Cu and CoFe/Cu multilayer giant magnetoresistive (GMR-ML) sensors with high thermal stability and high linearity using the Ultra-High Vacuum Magnetron Sputtering technique and successfully applied them to medical ventilator current sensors [17]. However, the GMR sensors are unipolar and cannot detect current direction without an external bias. The spin valve and TMR sensors are bipolar and sensitive to both the strength and direction of the magnetic field, as well as having advantages of small size, high sensitivity, high-frequency response, anti-interference, and low power consumption, so they are well-suited for designing current sensors. The TMR sensors combined with a magnetic loop can achieve a high sensitivity of 10 V/A, and the minimum error is only 0.22%, as shown by a design detecting ±200 mA small currents in power systems [22]. T. C. Gawade et al. developed a noninvasive low-current sensor with a spin valve multilayer membrane structure [23]. In sensor design, P. Ripka’s work has created a sensor that does not require a magnetic ring, making the sensor lighter and free from hysteresis [24]. However, the current sensors with magnetic rings are better at shielding external interference, offering greater precision for high-accuracy measurements.

Current sensors are usually designed to place the sensing chip in the air gap, which makes the sensing chip easy to quickly saturate [25], especially for high-sensitivity sensors with a relatively small linear operating range. This limits the range of current measurement. We propose an open-loop current sensor design to address this issue. By utilizing the induction effect of the soft magnetic ring on the magnetic circuit, the magnetic field generated by the signal current can be effectively attenuated. This helps maintain the spin-valve sensing chip within its linear operating range, expanding the current detection range. This design combines spin sensing technology, soft magnetic shunt technology, and multi-sensor data fusion, using four high-sensitivity spin valve sensor chips to detect the magnetic field. The soft magnetic shunt structure shunts and attenuates the strong magnetic signal generated by high currents, extending the sensor’s detection range. Moreover, this paper also improves the system’s robustness by combining multiple sensor chips with the mean data fusion algorithm, which makes up for the possible deviations in the sensor’s fabrication process and imprecise installation of current-carrying wires.

## 2. Design and Simulation of the Current Sensor

### 2.1. Principles and System Design

**Figure 1:**
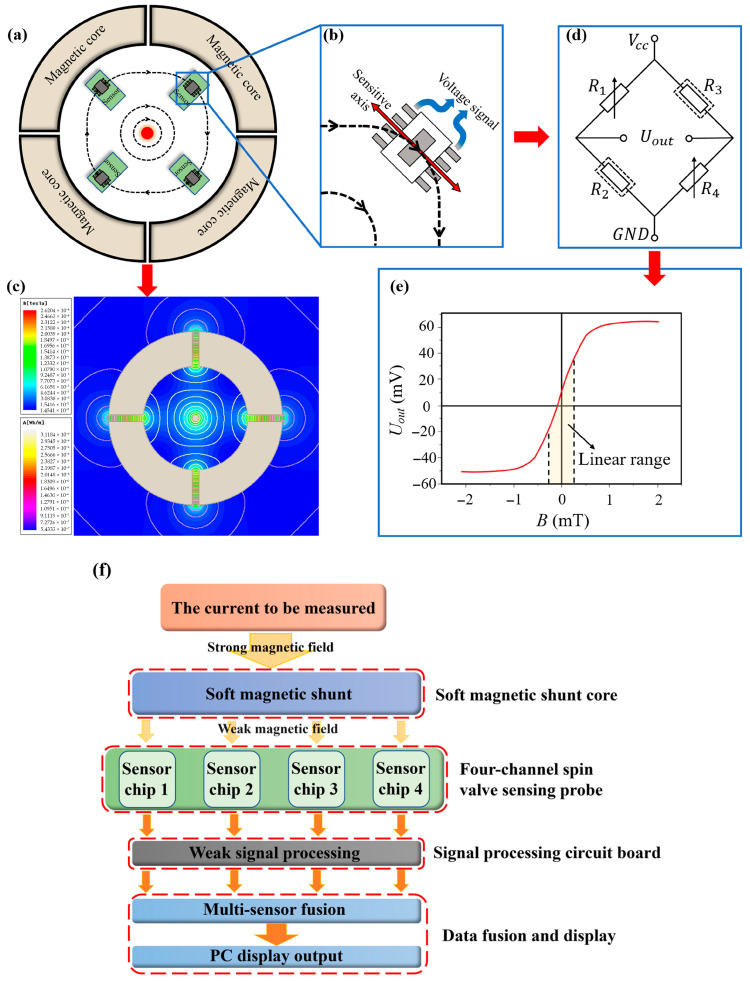
(**a**) Schematic structural model of current sensor based on spin sensing and magnetic shunt structure. (**b**) Alignment of the spin valve sensor chips’ sensitive axis with the magnetic induction intensity direction. (**c**) Shielded Wheatstone bridge configuration. (**d**) Simulation results of magnetic induction intensity distribution. (**e**) Input–output curve of the spin valve sensor chip. (**f**) Schematic illustration of the current sensor’s operational workflow.

The magnetic ring structure guides the magnetic circuit, and its design influences the magnetic field distribution. In traditional designs, a single air gap is usually used to concentrate the magnetic field to increase the magnetic induction and the sensor’s output signal. However, this often limits the sensor’s current detection range. To solve this problem, we propose a four-air-gap configuration in the magnetic ring (Figure 1a) to extend the detection range.

The magnetic core is an iron-based amorphous material with high saturation magnetization, high permeability, and low hysteresis loss [26]. The soft magnetic shunt core is composed of four symmetrically distributed annular segments, with four spin valve sensors positioned in the magnetic attenuation zone. Each spin valve sensor chip aligns with the tangential direction of the magnetic field lines at an angle of 45° to the air gaps and senses the magnetic induction intensity and outputs a weak differential voltage signal, as shown in Figure 1b. The magnetic simulation results show that the soft magnetic core forms a magnetic field attenuation zone, reducing the magnetic induction intensity at the sensor locations compared with the air gap (Figure 1c).

The spin valve sensor chip is crucial for detecting the magnetic field generated by the signal current. In this paper, we use the self-developed SAS030-1 spin valve sensor chip. This chip adopts a shielded Wheatstone bridge structure, as shown in Figure 1d. It consists of four identical magnetoresistive units, which can minimize temperature drift and detection errors, and ensure high sensitivity and small size [23,27]. The chip has bipolar detection, high sensitivity, and accurate current measurement capabilities, and can measure the direction of the current. Figure 1e shows the input–output curve of the SAS030-1 chip. The chip operates at a 5 V supply voltage and responds to bidirectional magnetic fields within ±0.50 mT. The sensitivity is approximately 33 mV/V/mT, and the linearity is 0.5%. The spin valve sensor chip has good thermal stability with a temperature coefficient of 0.0001%/°C.

The open-loop current sensor system mainly includes the soft magnetic shunt core, four-channel spin-valve sensing probe, weak signal processing circuit, and data fusion and display module. As illustrated in Figure 1f, the current generates a strong magnetic field, which is then decayed into a weak magnetic field through the soft magnetic shunt. The four spin valve sensor chips simultaneously detect the magnetic field signal and output weak differential voltage signals, which are processed and fused to provide an output voltage signal reflecting the measured current.

The selection of the number of sensors involves a trade-off between system performance and cost. With only two sensors, placed on opposite sides, measurements would be significantly impacted by the direction of wire offset, resulting in substantial measurement errors. In contrast, the four-sensor configuration, combined with data fusion, can effectively reduce errors induced by wire displacement. Although adding more sensors (e.g., eight) could further improve accuracy, it would also increase both hardware costs and system complexity. Therefore, a four-sensor configuration is sufficient to achieve the desired performance within the ±5 A linearity range.

### 2.2. Experimental Simulation Settings

The Ansys Maxwell software (ANSYS Maxwell 16.0) is applied for the simulation and optimization of the current sensor design. The current-carrying wire material is set as “copper”, and the magnetic core is selected from the built-in “mu_metal” in the simulation platform. Both the air gap and attenuation layer are set as “vacuum”. The simulation region is defined using an adaptive sizing method, with the maximum element length of 2 mm.

### 2.3. Design and Simulation of Magnetic Shunt Structure

When simulating the structure of the soft magnetic shunt core, it is crucial to consider the interrelationships between the current-carrying wire, magnetic ring, spin valve sensor chip, and the current sensor’s detection range and accuracy. The material, size, and positioning of the wire, along with the magnetic core properties, determine the core’s structural dimensions.

In this study, the design range of the current sensor is set to ±5 A. Copper, selected for its excellent conductivity, is used for the current-carrying wire, with a radius of 1 mm. The wire’s maximum current capacity is calculated using the formula

(1) $$I_{MAX}=J_{MAX}\times A,$$

where $I_{MAX}$ is the maximum current the wire can withstand, $J_{MAX}$ is the maximum current density, and A is the wire’s cross-sectional area. The calculated maximum current $I_{MAX}$ for the copper wire is 15.7 A, well above the ±5 A range, ensuring a sufficient safety margin.

**Figure 2:**
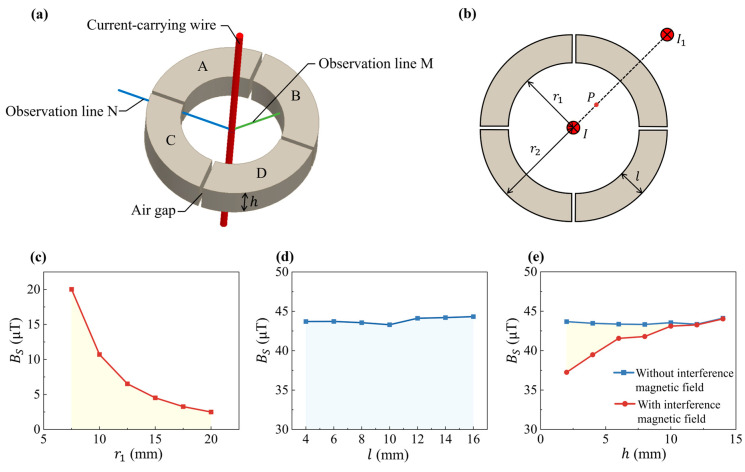
(**a**) Simulation model of magnetic shunt structure. (**b**) Schematic of the simulation model of the magnetic shunt test position P with external interference current $I_{1}$. (**c**) Influence of the inner radius $r_{1}$ of the magnetic ring on the magnetic induction $B_{S}$. (**d**) Effect of ring width l on the magnetic induction $B_{S}$. (**e**) Impact of ring height h on $B_{S}$ with or without interference magnetic field.

The soft magnetic core is made of an iron-based amorphous material with a maximum permeability of 450,000, a saturation magnetic induction of 1.56 T, a coercivity of less than 2.5 A/m, and a Curie temperature of 560 °C. The core’s design is shown in Figure 2a,b, where “A, B, C, D” represent the four magnetic core segments. The core’s dimensional parameters must balance various performance indicators. For instance, increasing the magnetic ring’s radius reduces magnetic induction at a given distance, extending the sensor’s measurement range, but also increases its size. This trade-off is essential to optimize both range and compactness.

During the simulation, we first keep the width *l* constant at 10 mm while changing the inner radius $r_{1}$. Simulation results in Figure 2c show that as the inner radius $r_{1}$ increases from 7.5 mm to 20 mm, the magnetic induction $B_{S}$ in the shunt region at a point 2 mm away from the inner wall of the magnetic ring decreases from 20.0 μT to 2.5 μT under a current of 1 A. By utilizing the rapid attenuation of $B_{S}$ as $r_{1}$ increases, we can design a magnetic ring with larger $r_{1}$ to obtain a wider detection range for the current sensor. This is one of the advantages of the current sensor based on magnetic shunt effect, compared to the traditional air-gap current sensor. In addition, we maintain the average value of the inner and outer radius at 20 mm and change the width of the magnetic ring (l). The width (l) primarily affects the magnetic flux density inside the ring, with little impact on the shunt region’s magnetic induction (Figure 2d). Taking into account that the width *l* will affect its mechanical strength, as well as its volume and weight, we have chosen a moderate value 10 mm. A gap that is too wide enhances the leakage field, introducing harmonic components [27], while a narrow gap increases manufacturing complexity and measurement errors. A 1 mm gap width is chosen for optimal field uniformity and feasibility. Besides the flux-shunting effect, the magnetic core can effectively shield external magnetic field interference to improve the sensor’s anti-interference ability. Simulation results in Figure 2b show that with a 1000 A interference current, the interference magnetic induction at P would be 3.75 mT without the magnetic ring. The shielding effectiveness is evaluated by comparing the magnetic induction at P with and without the interference field. The results in Figure 2e show significant shielding improvement when the magnetic ring *h* exceeds 10 mm. However, if *h* is too large, it will lead to excessive volume and weight. Consequently, the final magnetic ring parameters are $r_{1}$ = 15 mm, l = 10 mm, and h = 10 mm.

**Figure 3:**
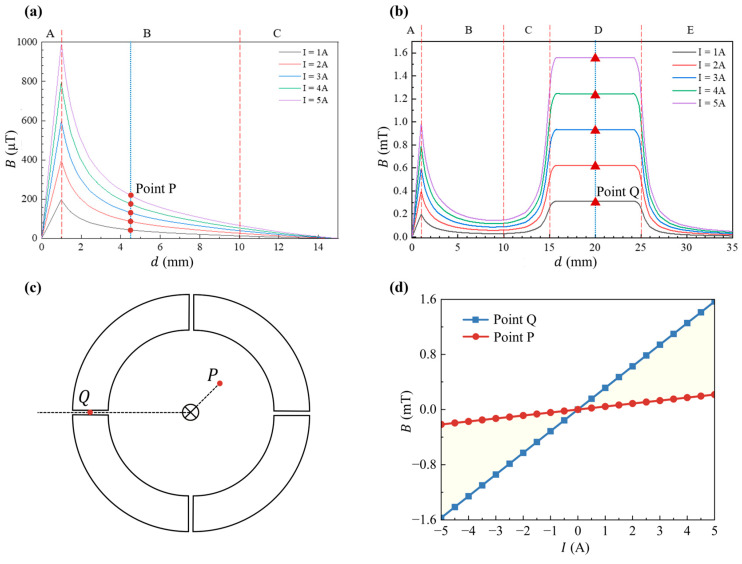
(**a**) Simulation results of *B*–*d* curves along the observation line M. (**b**) Simulation results of *B*–*d* curves along the observation line N. (**c**) Schematic diagram of the position of point P and point Q. (**d**) Simulation results of *B*–*I* curves at points P and Q.

Furthermore, we simulated and analyzed the magnetic induction intensity along two typical observation lines to study the magnetic shunt effect of the magnetic core. Observation line M extends from the wire’s center to the inner wall of the ring at the mid position of two adjacent air gaps, while line N runs from the wire’s center to the center of an air gap, extending 10 mm outward from the ring. The simulation results are presented in Figure 3.

### 2.4. Simulation of the Magnetic Shunt Effect

As shown in Figure 3a, the observation line M is divided into three intervals: A (0–1 mm), B (1–10 mm), and C (10–15 mm). Interval A spans from the wire’s center to its surface, while B and C represent the regions beyond the magnetic shunt. The signal current varies from 0 to 5 A in 1 A increments, corresponding to the five curves in Figure 3a. The magnetic induction along line M is strongest at 1 mm (the wire surface), reaching nearly 1000 μT. In intervals B and C, the magnetic induction *B* tends to decrease with the distance *d*, approaching 0 mT at the end of interval C, as expected, due to the magnetic core’s strong field aggregation near the ring [28]. In interval B (4–9 mm), the magnetic induction changes gradually, staying below 0.24 mT, well within the spin valve sensor chip’s linear range of ±0.30 mT, making it an ideal location for the spin sensor chip. In contrast, the 1–4 mm range in B experiences rapid changes exceeding the chip’s linear range, while the 9–15 mm region in C shows minimal magnetic induction, reducing the sensor’s sensitivity.

The simulation results for line N are shown in Figure 3b. Line N is divided into five intervals: A (0–1 mm), B (1–10 mm), C (10–15 mm), D (15–25 mm), and E (25–35 mm). Interval A covers the area from the wire center to its surface, while B and C correspond to the magnetic shunt region in the magnetic ring. Interval D represents the air gap across the core, and E is the region outside the soft magnetic core. The magnetic induction *B* on line N varies from line M. In interval B, the magnetic induction *B* decreases slowly, while as interval C approaches the air gap, *B* increases rapidly. For a current of 5 A, at 15 mm near the air gap, *B* reaches 1.200 mT, caused by the leakage field at the air gap. In interval D, *B* further increases to 1.570 mT, and in interval E, *B* decreases with distance until approaching 0 mT.

To ensure that the magnetic field intensity remains within the spin-sensing chip’s linear range (±0.300 mT) when the signal current is in the range of −5 A and 5 A, we selected a point 4.5 mm from the wire center (point P, Figure 3c) to install the spin-valve sensing chips. The simulation results show that there is a linear relationship between the magnetic induction intensity *B* and the signal current *I* at this point, and the ratio of *B*–*I* is 0.044 mT/A (Figure 3d). At 5 A, the value of *B* is 0.217 mT, well within the chip’s linear range, ensuring accurate measurement.

In addition, simulation at 20 mm from the wire center (point Q, Figure 3c) shows a *B*–*I* ratio of 0.314 mT/A, with *B* reaching 1.570 mT at 5 A, which is beyond the chip’s linear range. When the chip works in the maximum linear range (0.300 mT), the maximum measurable current is 6.8 A. These results provide a solid theoretical foundation for the current sensor’s design and current range selection in subsequent experiments.

Due to the magnetic shunt effect, positions farther from the conductor center could theoretically achieve even greater range extension. For example, for the position 1 mm from the inner wall of the magnetic ring (14 mm from the conductor center), *B* is about 2.2973 µT for a current of 1 A. This indicates that the range can be extended to about 130 A. However, for the selection of the detection point, it is not always optimal to simply pursue a larger range. First, due to the decrease in *B*, increasing the range means simultaneously reducing the sensitivity. Thus, a balance must be struck between extending the range and maintaining higher sensitivity. Additionally, in practical assembly, there are dimensional constraints on the sensor probe and signal processing circuit, which necessitate leaving appropriate installation space near the inner wall of the magnetic ring.

### 2.5. Performance Simulation in the Case of Imprecise Installation

Maintaining precise wire positioning during installation is challenging, often leading to significant deviations in single-sensor measurements. To address this, a multiple spin valve sensor approach combined with data fusion is employed to enhance the measurement stability and accuracy.

**Figure 4:**
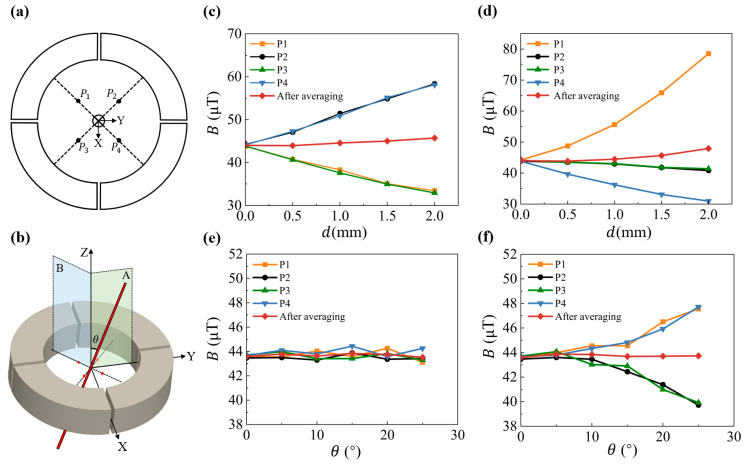
(**a**) Schematic of the wire displacement simulation model. (**b**) Schematic diagram of wire tilt simulation model. (**c**) Simulation results for wire displacement of the along the Y-axis. (**d**) Simulation results for the wire displacement relative to P1. (**e**) Simulation results for wire tilt in plane A. (**f**) Simulation results for wire tilt in plane B.

To verify the effectiveness of this method under various imprecise wire installation conditions, wire displacement and tilt are modeled in two representative simulation scenarios, with each, respectively, divided into two types. Wire displacement (Figure 4a) comprises translational displacement along the Y-axis (between measurement points) and translational displacement relative to a specific point (e.g., P1). Wire tilt (Figure 4b) includes tilt along the Y-axis (in plane A) and tilt toward a specific point (in plane B). The decomposition is based on the physical plausibility that a wire offset in 3D space can be represented by translational displacement along two directions and tilt around two axes. The chosen scenarios effectively capture typical wire position changes, making the simulation both universal and representative. For the wire offset simulation, a current of 1 A is applied. The wire is translated along the Y-axis, and changes in *B* at four test points (P1, P2, P3, and P4) are observed. As shown in Figure 4c, the signals at P2 and P4 increase while those at P1 and P3 decrease. Relying on data from a single-sensor results in a large error (32.61% at 2 mm), but this can be reduced by averaging the data from all four sensors through data fusion, and the averaging expression is

(2) $$U_{avg}=\frac{1}{4}\sum_{i=1}^{4}U_{i}$$

where $U_{i}$ is the voltage measured by each sensor. After sensor fusion, the error is reduced to 3.88%. Displacing the wire toward a specific measurement point (e.g., P1) results in an even larger error when using only P1’s measurement, as shown in Figure 4d. For example, a 2 mm displacement causes an error of 78.56%. However, multi-sensor data fusion can reduce the error, improving measurement accuracy, with the error dropping to 8.95%. This demonstrates that the method can reduce the error by an order of magnitude. In actual assembly, the alignment holes in the center of the probe board, signal-processing board, and base, ensure that the installation error of the current-carrying conductor does not exceed 0.1 mm. Simulations indicate that the measurement error after data fusion remains below 0.22%.

Another critical factor affecting accuracy is wire tilt. As illustrated in Figure 4b, the current is 1A. When the wire is tilted relative to the Z-axis, the magnetic field distribution changes. Simulations for tilt within plane A and plane B are conducted. The simulation results are shown in Figure 4e,f. When tilted within the A-plane, the magnetic induction intensity *B* at each sensor remains relatively stable, and the averaged data are more stable than individual sensor readings. In contrast, tilting within the B-plane, points P1 and P4 experience increased intensity due to the perpendicular distance, while P2 and P3 decrease as their sensitive axes tilt away from the wire.

Generally, when the wire’s deviations are asymmetrically distributed relative to the four sensors, such as when the current-carrying wire is tilted by 5° in a plane that forms a 15° angle with plane A and displaced by 0.1 mm, the resulting error is initially 1.08%. However, after data fusion, the error is reduced to 0.27%, demonstrating the method’s effectiveness in minimizing errors under general conditions. These simulation results are specifically included to verify the effectiveness of the proposed four-sensor design and data fusion method in compensating for errors caused by displacement or tilt of the current-carrying wire, thereby directly addressing potential concerns regarding its practical applicability. This approach not only works well for the current sensor presented in this work but also applies to other scenarios involving significant displacement deviations.

Overall, multi-sensor data fusion significantly enhances the system’s robustness to installation errors, whether from translational displacement or tilt.

## 3. Hardware Circuit and Software Design

**Figure 5:**
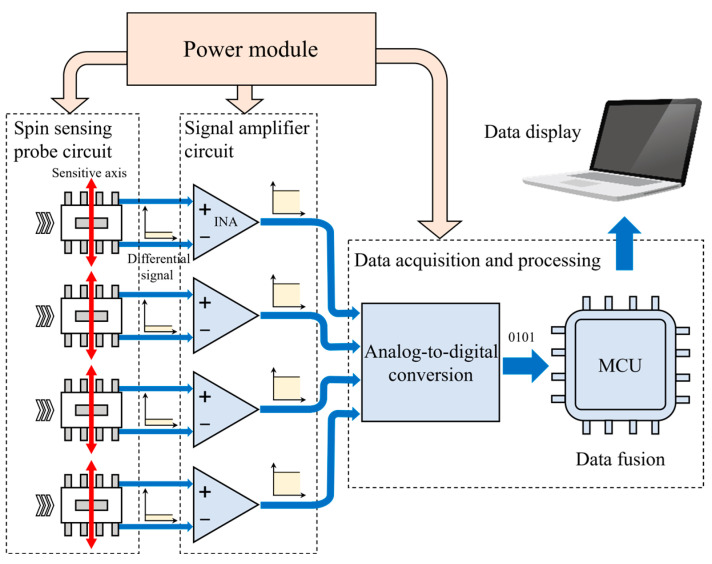
Schematic diagram of the signal processing flow of the current sensor.

**Figure 6:**
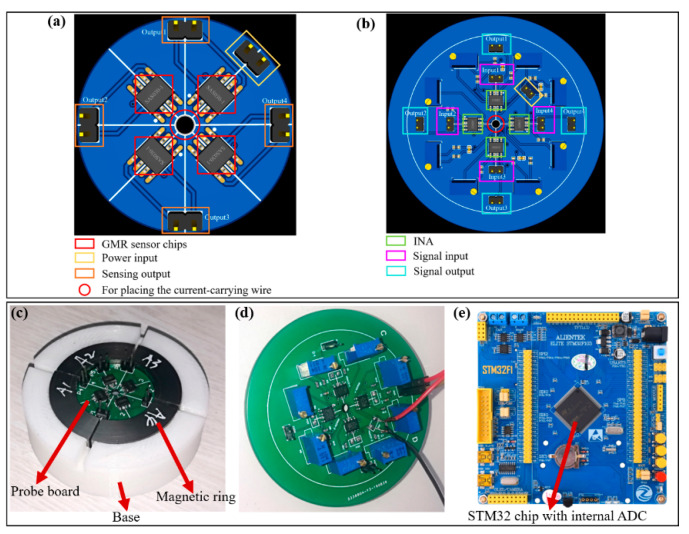
(**a**) Layout of the four-channel spin sensing probe circuit. (**b**) Layout of the signal amplifier circuit. (**c**) Assembly of spin sensing probe, magnetic ring, and base. (**d**) Photograph of the signal amplifier circuit and (**e**) the STM32 module.

The hardware circuit comprises four components: a four-channel sensor probe with spin valve sensor chips, a four-channel signal processing circuit, a multi-channel signal fusion and display circuit using STM32, and the power supply. The circuit schematic is shown in Figure 5. The spin valve sensor chips operate at 5 V, and upon detecting the external magnetic field, they generate a differential voltage via their Wheatstone bridges. Since the bridge output is only a few tens of millivolts, an amplification stage is required. The AD623 instrumentation amplifier amplifies the signal by a factor of 50, making it suitable for the ADC’s acquisition range. The amplified analog signals are then converted to digital form by the ADC, fused, and processed by the STM32, with the results displayed on a PC. The signal amplification circuit layout is shown in Figure 6b. The STM32, featuring three integrated ADCs, is used for data acquisition.

To ensure a secure connection between the sensor probe and the magnetic ring, we designed the PCB of the sensor probe with a circular shape. A 1 mm radius hole in the center allows for wire passage, as shown in Figure 6a,b. Figure 6c illustrates the actual assembly of the magnetic ring, base, and sensor probe. To enable external connections, five input/output connectors are placed along the edge of the circular PCB. This arrangement ensures stable hardware connections to the signal processing board, maintaining reliable signal transmission.

The layout and photograph of the signal amplification circuit are shown in Figure 6c,d. A 1 mm hole in the center of the board facilitates wire passage, and four amplifier output connectors on the outermost circle allow for signal output and processing. It should be noted that the magnetic ring provides effective shielding, minimizing external interference. Moreover, the magnetic ring is isolated from external interference with a minimum 10 mm distance, reducing the error to 0.11% even with a strong interference of 1 A current close to the air gap, in contrast to 76.85% without the magnetic ring, demonstrating the effectiveness of combining mechanical isolation with magnetic shielding in suppressing interference.

**Figure 7:**
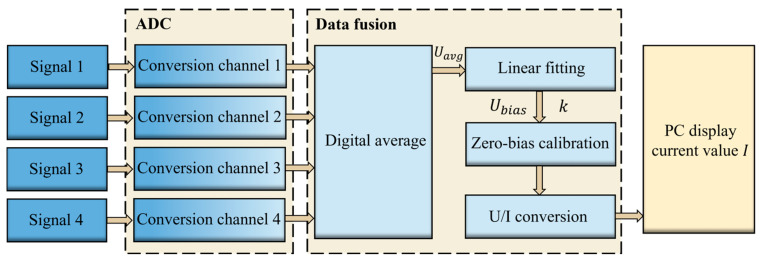
Block diagram of the multi-sensor data fusion algorithm flow.

The STM32’s internal 12-bit ADC module, with a 1 MHz conversion rate, 0.024% resolution, and a low power consumption, is used for synchronized signal acquisition from four sensors. The ADC scanning mode, combined with direct memory access (DMA), facilitates efficient and real-time data transfer. The multi-sensor data fusion process is shown in Figure 7. First, the amplified analog voltage signal of the sensor is converted into digital signal by ADC scanning, and the MCU acquires four digital voltage signals and calculates the four average signals $U_{avg}$ through Equation (2), and does a linear fitting to the four average signals to determine the zero-bias voltage $U_{bias}$ and the voltage-to-current conversion ratio *k*. The average signal $U_{avg}$ is first calibrated by subtracting the zero-bias voltage $U_{bias}$, and then converted into the measured current *I* through the ratio *k*. The derivation equation is

(3) $$I=\frac{U_{avg}-U_{bias}}{k}.$$

## 4. Results and Analysis

The current sensor’s sensitivity, linearity, and other key parameters were evaluated using the self-built test platform, comprising a regulated voltage source IT6332B, a bipolar output current source IT6121B, a high-precision Gaussmeter Lakeshore 425, and a digit multimeter 34411A. The voltage source provides a 12 V voltage for the relay on the bipolar output current source, which supplies −5 A to 5 A current for the current-carrying wire. The high-precision Gaussmeter measures the magnetic induction intensity, while the high-precision multimeter records the current sensor’s output voltage.

**Figure 8:**
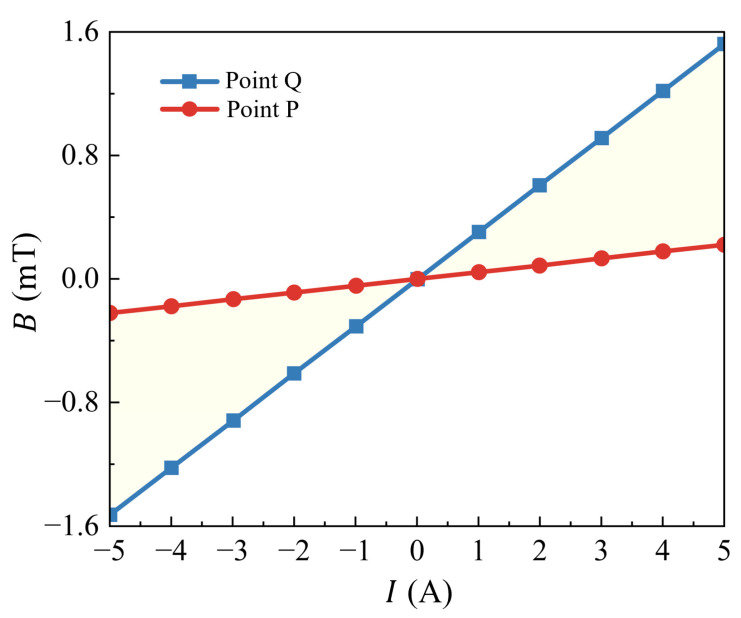
Test results of *B*–*I* curves at points P and Q.

We analyzed the variation in *B* with the current *I* at a point 20 mm from the wire center (point Q, located in the center of the air gap). As shown in Figure 8, *B* is proportional to *I* with a ratio of 0.305 mT/A, which is very close to the simulated value of 0.314 mT/A in Figure 3d. In addition, for current between −5A and +5A, *B* at the Q point fluctuates by approximately ±1.526 mT. This indicates that if the sensor chip is installed at this position, it is only suitable for measuring currents less than 1 A, not for full-range measurements of ±5 A.

Figure 8 shows the variation in magnetic induction intensity *B* at point P with current *I*. There is an excellent linear relationship, and the *B*–*I* ratio is 0.044 mT/A, matching the simulation results in Figure 3d. This verifies the accuracy of the simulation model and the design’s feasibility.

At point P, within the ±5 A current range, the magnetic induction intensity *B* changes from −0.221 mT to 0.221 mT, which meets the spin valve chip’s linear operation requirements. Compared with point Q, the magnetic induction at P is about 6.9 times smaller, increasing the sensor’s measurable current range from 1 A to 6.9 A. By combining the soft magnetic shunt with the sensitive spin-sensing chip, not only is the sensitivity improved, but also, the measurement range is extended.

**Figure 9:**
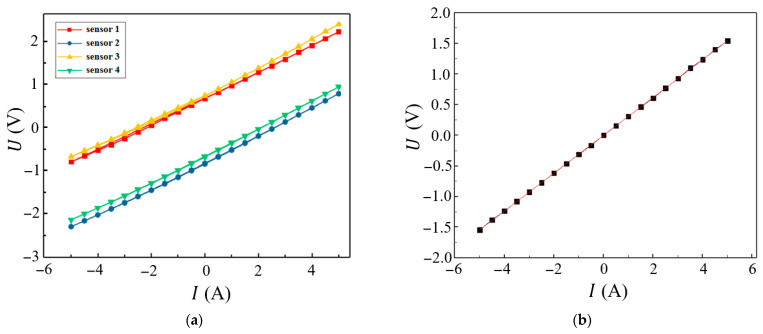
Comparison between before and after fusion of multiple-sensor data: (**a**) *U*–*I* curves of four spin valve sensors, (**b**) *U*–*I* curve after multi-sensor data fusion.

After calibrating the spin-sensing chip’s zero bias, we tested the relationship between the output voltage and the current *I* of the four spin sensors, as shown in Figure 9a. The relationship curves of the four-channel sensor are linear, but each sensor exhibits different biases due to installation deviations. The key parameters for the four spin valve sensor chips are listed in Table 1.

Although the sensitivity is similar, the bias difference is significant, which makes it difficult to determine which sensor has the most accurate measurement.

Compared to other methods, such as loop integration, the averaging algorithm is relatively simple and already effective enough to reduce the errors caused by conductor displacement.

We averaged the data of the four sensors and performed linear fitting (Figure 9b) to obtain a *U*–*I* ratio of 0.308 V/A for the current sensor at room temperature. With a 5 V power supply, the current sensor sensitivity was 61.6 mV/V/A, and the linearity was 0.55%. The calculated *B*–*I* ratio at the sensor’s location was 0.034 mT/A, close to the simulation value in Figure 3d, further confirming the design’s feasibility.

The system linearity (0.55%) is very close to the intrinsic linearity (0.5%) of the spin-valve chip, indicating that the nonlinearity primarily originates from the chip’s inherent characteristics. The performance of the designed current sensor is exceptional, as the design optimizes linearity by considering multiple factors. This includes the use of low-hysteresis iron-based amorphous materials and an optimized magnetic shunt structure, which ensures that the spin valve chip operates within its linear range with sufficient margin. High-linearity chips are selected carefully for the signal processing circuit, while multi-sensor data fusion further reduces nonlinear errors.

The data fusion effectively reduced the measurement deviations, eliminated the zero drift, and minimized the errors caused by external vibrations or changes in the current-carrying wire’s position.

To further assess the sensor’s temperature characteristics, we measured the sensitivity temperature coefficient of the system’s four-channel outputs. The sensitivity temperature coefficients for the four channels were −0.063%/°C, −0.084%/°C, −0.131%/°C, and −0.038%/°C, respectively.

To verify the system’s stability under real operating conditions, the output voltage of the sensor was recorded at a full-scale current of 5 A over a 6 h period, and the output drift error remained within 0.676%, demonstrating a good thermal stability.

In practical applications, conductors may not always be perfectly straight, which can lead to asymmetric magnetic field distributions. However, our magnetic-ring design and data fusion method can effectively mitigate these effects to ensure high accuracy.

To better highlight the contribution of this work, we compare our current sensor with similar sensors [29,30,31,32], as summarized in Table 2. Compared to other current sensors, ours demonstrates relative advantages in certain key parameters such as linearity, sensitivity, or measurement range.

## 5. Conclusions

This study proposes a novel type of open-loop current sensor that combines spin sensing, soft magnetic shunt technologies, and multi-sensor data fusion. The soft magnetic shunt significantly attenuates the magnetic field, expanding the current detection range. This detection range can be extended 6.8 times compared to the ring air gap range. A multi-sensor data fusion approach is introduced to improve measurement accuracy and reduce errors from installation misalignment. This method utilizes the data of four sensing chips to compensate for the mean difference, effectively minimizing the errors caused by wire displacement and tilt, thereby enhancing the sensor’s stability and robustness in practical applications. The test results show the sensor’s sensitivity is 61.6 mV/V/A, and the linearity is 0.55%, which ensures high-precision current detection. Furthermore, simulations and experimental results validate the magnetic shunt effect’s effectiveness, with a measured *B*–*I* ratio of 0.034 mT/A, aligning closely with theoretical expectations. The fusion algorithm reduces measurement errors from 78.56% to 8.95% under wire displacement conditions and from 1.08% to 0.27% in combined tilt/displacement scenarios, demonstrating robustness. Thermal drift remains below 0.676% over six hours, and external interference (1 A) is attenuated to a 0.11% error, verifying the system’s stability in complex environments. The developed current sensors can be widely used in standard devices, high-precision power metering devices, and high-voltage power distribution systems, providing reliable technical support for high-precision and large-range current measurements in the future. Future work will aim to integrate this design based on multiple sensors and the magnetic shunt effect with closed-loop compensation methods to further improve system linearity, bandwidth, and dynamic response.

## Figures and Tables

**Table 1:** Parameters for the four spin valve chips.

Characteristics	*U*–*I* Ratio (V/A)	Bias (V)	Linearity (%)
Chip 1	0.259	1.046	0.370
Chip 2	0.298	0.354	0.451
Chip 3	0.330	1.331	0.770
Chip 4	0.304	0.020	0.230

**Table 2:** Comparison of key parameters of this work with other similar current sensors.

Characteristics	[29]	[30]	[31]	[32]	This Work
Sensing principle	TMR	TMR	Hall	TMR	Spin valve
Linearity	0.95%	0.8%	1%	0.3%	0.55%
Characteristics	Bipolar	Bipolar	Bipolar	Unipolar	Bipolar
Sensitivity (mV/V/A)	-	-	80	30.3	61.6
Range (A)	±3	±15	±5	20	±6.8

## Data Availability

Data are contained within the article.
